# Smoking exposure, loss of forced expiratory volume in one second and the risk of lung cancer among patients with malignant disease who present with cardiac or pulmonary symptoms: a cross-sectional study

**DOI:** 10.1186/s12971-017-0122-2

**Published:** 2017-03-09

**Authors:** Siegfried Wieshammer, Jens Dreyhaupt

**Affiliations:** 1 Department of Cardiology, Pulmonology and Critical Care Medicine, Offenburg Hospital, Weingartenstrasse 70, D-77654 Offenburg, Germany; 2 Institute of Epidemiology and Medical Biometry, University of Ulm, Schwabstrasse 13, D-89075 Ulm, Germany

## Abstract

**Background:**

Smokers with airway obstruction are at a higher risk of lung cancer than smokers without airway obstruction. Inflammation plays a key role in lung carcinogenesis. This single-center study prospectively assessed *(i)* the relationship between smoking exposure and the loss of forced expiratory volume in 1 s (FEV1) in determining lung cancer risk and *(ii)* the effect of lung cancer on systemic inflammation.

**Methods:**

The study group comprised 475 consecutively enrolled patients with cancer who presented with pulmonary or cardiac symptoms. The effects of smoking exposure and FEV1 loss on the predicted lung cancer risk were assessed using multiple logistic regression analysis. C-reactive protein (CRP) was used as a marker of inflammation.

**Results:**

The prevalence of lung cancer was 0.23. The lung cancer risk increased with the number of pack years and FEV1 loss (*p* < 0.01). Moving from the 5^th^ (−22% of the predicted value) to the 95^th^ percentile of FEV1 loss (56% of the predicted value) increased lung cancer risk from 0.07 to 0.23 (Δ = 0.16) at 0 pack years and from 0.39 to 0.73 (Δ = 0.34) at 70 pack years (95^th^ percentile). The values for Δ peaked at 61 pack years (0.34) and then decreased with a further increase in smoking exposure, without reaching the zero mark. Patients with lung cancer were more likely to have a CRP level above the median (4.05 mg/L) than patients with other cancers (adjusted odds ratio = 2.67).

**Conclusions:**

Systemic inflammation is more pronounced in patients with lung cancer than in patients with other cancers. The effect of FEV1 loss on the patients’ predicted risks of lung cancer increases with increasing smoking exposure. Measurements of FEV1 loss are useful to identify patients facing an increased risk of developing lung cancer.

## Background

Chronic obstructive pulmonary disease (COPD) and lung cancer co-occur in smokers more frequently than if they were independently caused by smoking. According to epidemiological studies, the lung cancer risk increases with decreasing forced expiratory volume in 1 s (FEV1) after adjusting for the smoking dosage [1, 2]. As reported in a meta-analysis, even a slight decrease in the FEV1 from > 100 to 90% of the predicted value increased the adjusted risk for lung cancer 1.30-fold in men and 2.64-fold in women [3]. These risks were increased by 2.23-fold in men and 3.97-fold in women exhibiting an FEV1 of 70% of the predicted value. In addition to smoking exposure, several mechanisms may account for the link between lung cancer and FEV1 loss, including a genetic overlap between both entities. Shared genetic pathways to disease development may predispose the small airways of susceptible smokers to oxidative stress and the accumulation of inflammatory cells. Fibrosis, inflammation of the small airways and the presence of luminal exudates are both features of COPD and carcinogenic risk factors. The extents to which these processes occur correlate with the rate of FEV1 loss [4]. Therefore, FEV1 loss may be considered a quantitative surrogate marker of the smoking-induced carcinogenic damage to the airways. The purpose of this study was to further delineate the relationship between smoking exposure and FEV1 loss in determining patients’ risks of lung cancer. Secondary objectives were to determine *(i)* whether patients with lung cancer differ from patients with other cancers with respect to the degree of systemic inflammation, and *(ii)* whether the effect of smoking exposure on FEV1 loss differs in patients with lung cancer compared to patients with other cancers.

## Methods

### Patients

**Fig. 1:**
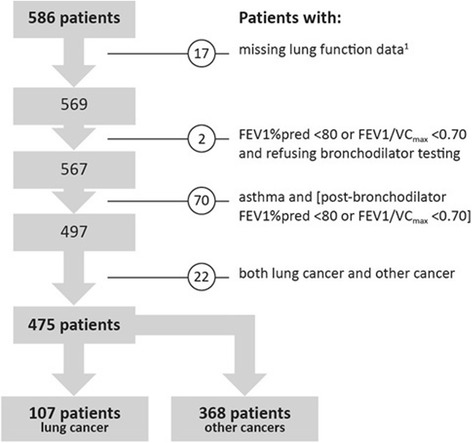
Patient selection algorithm. ^1^The patient did not turn up for lung function testing (*n* = 2), was deemed too sick for spirometry (*n* = 4), or was deemed too sick to sit in the plethysmograph, but the TLC % predicted value was required for adjusting the FEV1 % predicted value in patients with restrictive lung disease (*n* = 11)

This prospective single-center study included 586 consecutively enrolled patients with a history of either previous or active malignant disease who were referred from primary care, oncology, or radio-oncology to the pulmonology or cardiology service of an academic teaching hospital between May 2007 and October 2014 because of dyspnea, cough, chest pain, pulse irregularities, or exercise intolerance. All but 8 patients were outpatients, and none were confined to bed. We excluded 111 patients for the reasons presented in Fig. 1, resulting in the inclusion of 475 patients in the analysis.

### Diagnostic procedures

The patients underwent an examination to identify lung and heart disease that included a medical history, smoking history, chest X-ray and spirometry, as well as an electrocardiogram and an echocardiogram, as previously described [5]. Further tests were performed when indicated. The European Respiratory Society predicted values for normal lung volumes were utilized [6]. Patients exhibiting an FEV1 < 80% of the predicted value or an FEV1/forced expiratory vital capacity (FVC) ratio < 0.70 and patients with a history or suspicion of bronchial asthma underwent bronchodilator testing with 400 μg of salbutamol from a metered dose inhaler via a Volumatic spacer (GlaxoSmithKline, Munich, Germany). The maximum values for FEV1 and vital capacity (VC) obtained either before or after bronchodilator testing were used for further analysis. Patients with a VC < 80% of the predicted value underwent body plethysmography to determine total lung capacity (TLC). In patients exhibiting both an FEV1 < 80% of the predicted value and a TLC < 80% of the predicted value, the FEV1 % predicted value was adjusted for the degree of restriction *(i)* by dividing the FEV1 % predicted value by [0.01 x TLC % predicted] if the TLC % predicted value was greater than the VC % predicted value and *(ii)* by dividing the FEV1 % predicted value by [0.01 x VC % predicted] if the VC % predicted value was greater than or equal to the TLC % predicted value. The lifetime active smoking exposure was measured in pack years. Assuming 20 cigarettes per pack, this value is equal to the number of packs of cigarettes smoked per day multiplied by years of consumption. A web-based pack year calculator (www.smokingpackyears.com/) that accounted for varying smoking habits over the years was used. The FEV1 loss % predicted was calculated as [100% - FEV1 % predicted].

The serum C-reactive protein (CRP) levels were measured using a highly sensitive assay (COBAS Integra, CRPLX, Roche Diagnostics, Mannheim, Germany). The lower limit of detection for the CRP levels was 0.71 mg/L. A value of 0.35 mg/L was prospectively assigned to 45 patients with undetectable CRP levels, as previously done in this cohort [5]. The estimated glomerular filtration rate (eGFR) was also calculated [7].

### Data analysis

Patients were stratified into three groups according to the level of FEV1 loss to determine whether even a slight FEV1 loss (0–20% predicted value) was associated with an increased risk of lung cancer; i.e., < 0% predicted (stratum 1), 0–20% predicted (stratum 2) and > 20% predicted (stratum 3). A univariate logistic regression analysis was used to evaluate the association, which was presented as an odds ratio (OR) and 95% confidence interval (CI), between FEV1 loss stratum membership and the presence of lung cancer. This association was tested in unadjusted and adjusted models that controlled for smoking exposure in pack years. Patients exhibiting an FEV1 loss of < 0% predicted formed the reference group.

A risk prognosis model based on multiple logistic regression analysis was used to evaluate the association between active smoking exposure and FEV1 loss with the risk of lung cancer. The difference in risk (Δ) at an FEV1 loss of 56% (95^th^ percentile) and −22% of the predicted values (5^th^ percentile) was normalized to the maximum possible increase in risk for a given smoking exposure as follows: Δ_norm_ = Δ/(1 – risk at an FEV1 loss of −22% of the predicted value). In a second step, the association of smoking exposure and FEV1 loss with the predicted risk of lung cancer was adjusted for age and sex.

The CRP levels displayed a skewed distribution and were transformed to their natural logarithms (ln) to achieve normality. A linear regression model was used to analyze the effect of lung cancer on ln (CRP) before adjustment and after the first adjustment for the following prospectively defined confounders: body mass index (BMI) [8], age [9], and eGFR [10] as continuous variables, and the smoking status at the time of referral (current *versus* never or ex-smoker) [11], sex [12], and the presence of active malignant disease or heart disease [13] (see Table 1 for definition) as categorical variables. In the second step, the values for ln (CRP) were additionally adjusted for FEV1 loss. The change in the standardized regression coefficient (β) was used to determine the extent to which the second adjustment attenuated the effect of lung cancer on ln (CRP) compared to the first adjustment. A sensitivity analysis was conducted to assess the effect of choosing a value of 0.35 mg/L for patients with undetectable CRP levels. This analysis compared the results obtained with 0.35 mg/L with those obtained with CRP values of 0.01 mg/L and 0.70 mg/L, respectively.

**Table 1:**
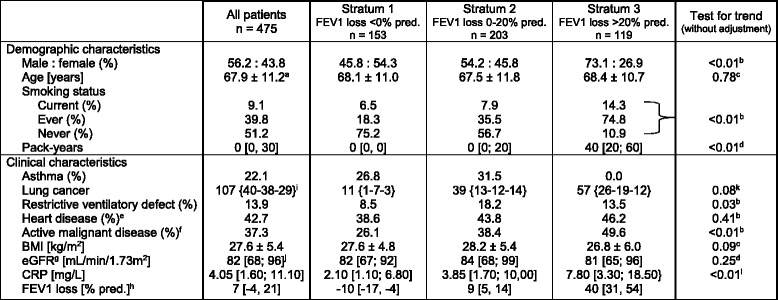
Demographic and clinical characteristics in the entire study group and in the three strata of FEV1 loss

^a^Mean value ± 1 standard deviation

^b^Chi square test

^c^One way analysis of variance

^d^Kruskal-Wallis test

^e^History of coronary artery disease defined as either angiographically proven coronary artery disease or a hospital diagnosis of myocardial infarction, presence of atrial fibrillation, impaired left ventricular systolic function (ejection fraction <50%), significant valvular heart disease, pulmonary hypertension, or left ventricular hypertrophy. Comprehensive definitions of these conditions are in given in reference 3

^f^Residual tumor at the time of referral or time interval between cancer surgery/end of chemotherapy/radiotherapy and referral <3 months

^g^Estimated glomerular filtration rate

^h^FEV1 loss [% predicted] = 100% - FEV1 % predicted

^i^Numbers of patients with {squamous cell cancer – adenocarcinoma – other histologic subtype}

^j^Median value, with interquartile range in brackets

^k^Fisher’s exact test, distribution of histologic subtypes

^l^One way analysis of variance for the log transformed values

The effect of smoking exposure on FEV1 loss was separately analyzed for patients with lung cancer and patients with other cancers using linear regression.

Given the exploratory nature of this study, the test results should not be interpreted as confirmatory, and no adjustments for multiple tests were performed. A *p*-value < 0.05 was considered significant. The analyses were performed using SAS version 9.4 (SAS Institute, Chicago, IL, USA).

## Results

Table 1 shows the demographic and clinical characteristics of the entire study group and their distribution according to the three strata of FEV1 loss. We observed 408 malignancies in the 368 patients with other cancers. Of the 40 patients with multiple tumors, 30 had different tumors. The most frequent entities were the following: breast cancer (*n* = 105), prostate cancer (*n* = 56), colorectal cancer (*n* = 37), and lymphomas (*n* = 70). Active malignant disease was present in 68% of the 107 patients with lung cancer and in 28% of patients with other cancers (*p* < 0.01).

The median [interquartile range] time intervals between cancer diagnosis and referral were 0.8 [0.3, 17.7] months for patients with lung cancer and 58.9 [19.5, 121.7] months for patients with other cancers (*p* < 0.01). The time interval between the cancer diagnosis and referral was > 5 years in 10% of patients with lung cancer and in 49% of patients with other cancers. Of the 29 patients with lung cancer who were diagnosed > 1 year before referral, none continued to smoke after receiving this diagnosis. The median time interval between the lung cancer diagnosis and referral was 42.0 [24.5, 116.5] months in this subgroup. Fourteen of the 29 patients exhibited an FEV1 ranging from < 80–50% of the predicted value, and 3 patients exhibited an FEV1 < 50% of the predicted value. The median time interval between the first cancer diagnosis and referral was 73.8 [39.1, 147.5] months in patients with other cancers who were diagnosed > 1 year prior to referral. Sixteen of these 308 patients were current smokers at the time of referral.

A restrictive ventilatory defect was present in 66 patients, 37 of whom had evidence of pulmonary restriction (parenchymal lung disease, 16 patients; prior lung resection surgery, 21 patients), and 19 had extra-pulmonary restriction due to the presence of pleural effusion (*n* = 8), pleural fibrosis (*n* = 5) or diaphragm paralysis (*n* = 6). Ten patients had no evidence of an underlying benign pleuropulmonary disease or diaphragm weakness.

**Fig. 2:**
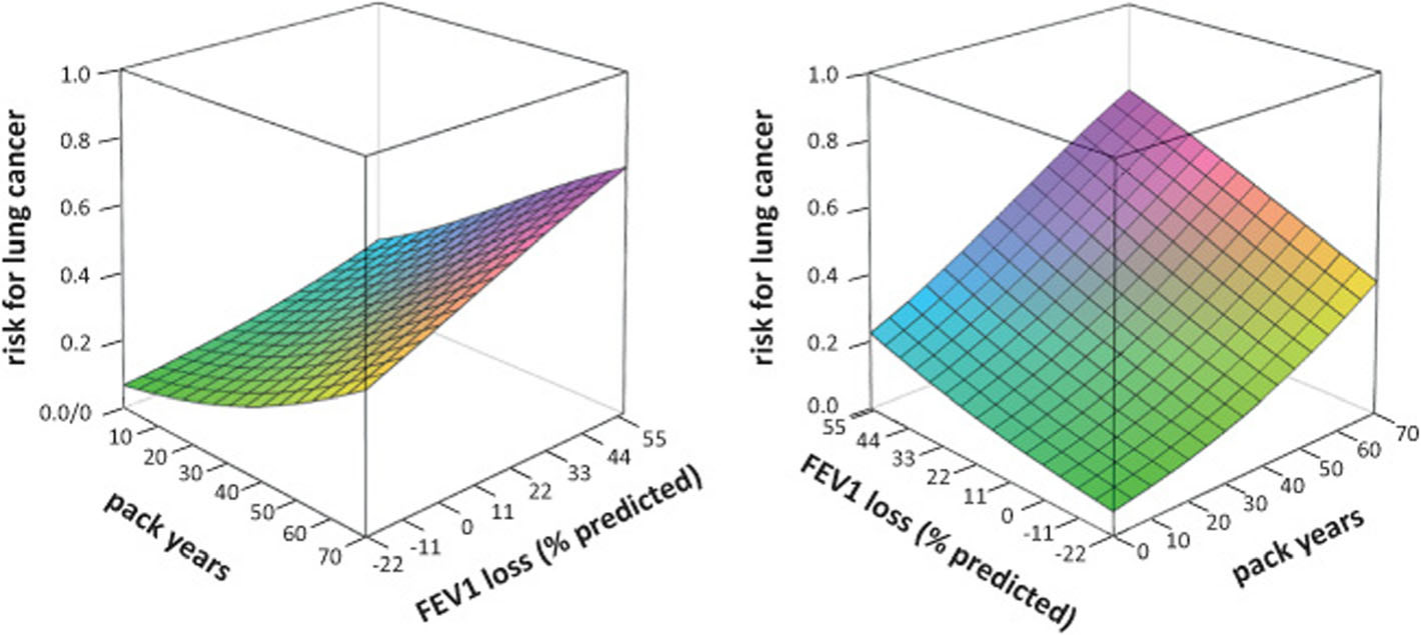
Impact of smoking exposure (pack years, x axis) and FEV1 loss (% of the predicted value, y axis) on the patient’s predicted risk of lung cancer (z axis, vertical axis) as determined by a risk prognosis model based on multiple logistic regression analysis. This model allows to estimate the patient’s risk of lung cancer (z) from the number of pack years (x) and FEV1 loss (y) using the following formula: $\mathrm{z}=\frac{1}{\left(1+{e}^{-\left(-2.2296+0.0312* x+0.0183* y\right)}\right)}$. The data from all patients were included in the regression analysis. The x and y axes of the figure were limited to 0 to 70 pack years (95^th^ percentile) and −22% to 56% of the predicted value (5^th^−95^th^ percentile), respectively, to minimize disproportionate effects of extreme values on the graph. The graph has a color code starting with green for a low predicted risk and ending with bluish-purple for a high predicted risk for lung cancer. A visualization tool that allows a three-dimensional display of the relationship between smoking exposure, FEV1 loss and the risk of lung cancer from various angles is available on the internet via http://www.ortenau-klinikum.de/fileadmin/resources/downloads/dr-wieshammer/smoking-exposure-3d-graph.exe

**Fig. 3:**
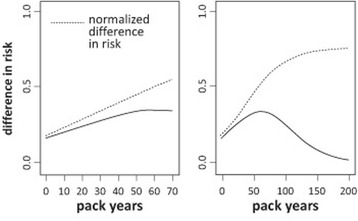
Differences between the risks of lung cancer (Δ; *solid lines*) at an FEV1 loss of 56% of the predicted value (95^th^ percentile) and −22% of the predicted value (5^th^ percentile) for smoking exposures from 0 to 70 pack years (95^th^ percentile; *left panel*) and over the whole range (0–200 pack years; *right panel*). The difference between the two risks (Δ) was normalized (normalized difference in risk; Δ_norm_; *dotted lines*) to the maximum possible increase in risk for a given smoking exposure using the formula: ${\Delta}_{\mathrm{norm}} = \frac{\Delta}{\left[1\hbox{--}\ \mathrm{risk}\ \mathrm{a}\mathrm{t}\ \mathrm{a}\ \mathrm{FEV}1\ \mathrm{loss}\ \mathrm{of}\ \left(-22\ \%\ \mathrm{of}\ \mathrm{t}\mathrm{he}\ \mathrm{predicted}\ \mathrm{value}\right)\right]}$

The patients exhibiting an FEV1 loss from 0 to 20% of the predicted value had a higher odds of lung cancer than patients assigned to the reference group before (OR 3.07, 95% CI 1.52–6.22) and after adjustment (OR 2.61, 95% CI 1.26–5.45) for smoking exposure. Both smoking exposure and FEV1 loss were associated with the predicted risk of lung cancer (*p* < 0.01), as illustrated in Fig. 2. The risk of lung cancer was 0.07 at 0 pack years if the FEV1 loss was at the 5^th^ percentile (−22% of the predicted value). Moving up the trajectory from the 5^th^ to the 95^th^ percentile for FEV1 loss (56% of the predicted value) while the smoking exposure was maintained at 0 pack years, increased the lung cancer risk to 0.23 (Δ = 0.16; Δ_norm_ = 0.18). At the 95^th^ percentile of smoking exposure (70 pack years), an increase from the 5^th^ to the 95^th^ percentile for FEV1 loss increased the risk of lung cancer from 0.39 to 0.73 (Δ = 0.34; Δ_norm_ = 0.55). The values for Δ increased from 0.16 at 0 pack years, to 0.24 at 20 pack years, to 0.31 at 40 pack years, and the values reached their maximum (0.34) at 61 pack years, followed by a decrease with a further increase in smoking exposure without reaching the zero mark, as illustrated in Fig. 3. This figure also shows that the graph Δ_norm_ *versus* number of pack years exhibited an almost linear increase from 0 to approximately 70 pack years and flattened out beyond this level of smoking exposure without reaching a plateau. The effects of smoking exposure and FEV1 loss on the predicted risk of lung cancer remained significant (*p* < 0.01) after adjustment for age (*p =* 0.48) and sex (*p* = 0.06).

Among the 107 patients with lung cancer (83 men and 24 women), 14 (1 man and 13 women) and 93 (82 men and 11 women) were never smokers and ever smokers, respectively. Squamous cell carcinoma was diagnosed in 40 patients (37%), followed by adenocarcinoma (*n* = 38; 36%) and other subtypes (*n* = 29; 27%). Ever smokers with an FEV1 loss > 20% of the predicted value had higher odds of lung cancer than smokers with an FEV1 loss that did not exceed 20% (OR 2.69, 95% CI 1.60–4.63). This odds ratio remained significant after adjusting for smoking exposure (OR 2.13, 95% CI 1.21–3.75). When restricting the analysis to the 203 patients who had a ≥ 10 pack-year history of smoking (36 [20–50] pack years), the patients with an FEV1 loss > 20% of the predicted value (*n* = 104; 40 [30–60] pack years) had a higher odds of lung cancer (OR 2.04, 95%-CI 1.16–3.58) than patients with an FEV1 loss ≤ 20% of the predicted value (*n* = 99; 30 [15–50] pack years).

The 177 patients with active malignant disease had higher (*p* < 0.01, *t*-test for log transformed values) CRP levels (8.40 [3.50–26.20] mg/L) than patients with inactive disease (2.50 [1.30–6.60] mg/L). The mean log transformed values for ln (CRP) were higher in the patients with lung cancer than in the patients with other cancers in the unadjusted analysis (2.40 *vs* 1.24). Patients with lung cancer were approximately four times more likely to have a CRP level above the median (4.05 mg/L) than patients with other cancers (OR 4.49, 95% CI 2.74–7.35). The effect of lung cancer on ln (CRP) remained significant (2.18 *vs* 1.49) after adjusting for BMI (*p* = 0.05), age (*n.s.*), eGFR (*n.s.*), smoking status at the time of referral (*p* = 0.02), sex (*n.s.*), the presence of active malignant disease (*p* < 0.01), and the presence of heart disease (*p =* 0.03). After the additional adjustment for FEV1 loss (*p* < 0.01), ln (CRP) and smoking status at the time of referral were no longer associated (*p* = 0.63), whereas the effects of BMI (*p* = 0.02), the presence of active malignant disease (*p* < 0.01), and the presence of heart disease (*p* = 0.04) on ln (CRP) persisted. The second adjustment for FEV1 loss attenuated the effect of lung cancer on ln (CRP) by 15.2%, as indicated by the change in β for lung cancer (0.197 *vs* 0.167). In the fully adjusted model, the values for ln (CRP) were still higher in the patients with lung cancer than in patients with other cancers (2.09 *vs* 1.51). The former group still had a nearly three-fold higher risk of having a CRP level above the median (OR 2.67, 95% CI 1.50–4.75).” Lung cancer” was compared to the other covariates to illustrate the magnitude of the effect of this predictor variable on ln (CRP). In the fully adjusted model, “lung cancer” had a 2.19-fold greater effect on ln (CRP) than “heart disease” and the same effect as an increase in FEV1 loss of 47% of the predicted value. Choosing a CRP value of 0.35 mg/L for patients with undetectable CRP values had a negligible effect on the results of the regression analyses and no effect on the CRP values presented in the table.

**Fig. 4:**
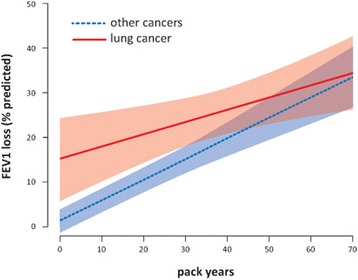
Relationship between smoking exposure and FEV1 loss (% of the predicted value) with 95% CIs as shaded bands around the trend lines in patients with lung cancer (*solid lines*) and other cancers (*dotted lines*) from the 5^th^ (0 pack years) to the 95^th^ percentile (70 pack years) of the predictor variable

The effect of smoking exposure on FEV1 loss differed in patients with lung cancer compared to patients with other cancers. Figure 4 shows that the slope of this relationship was higher in patients with other cancers (4.6% predicted per 10 pack years *vs* 2.7% predicted per 10 pack years, *p* = 0.03), whereas the baseline level at 0 pack years was higher in patients with lung cancer (15.2% *vs* 1.3% predicted, *p* < 0.01). The 95% CIs of the two curves began to overlap at approximately 30 pack years. The estimated crossover point was at 74 pack years, corresponding to an FEV1 loss of 36% of the predicted value.

## Discussion

The principal findings of this study are that FEV1 loss is a predictor of lung cancer risk after controlling for smoking exposure and that this effect of FEV1 loss increases with increasing smoking exposure. A FEV1 loss of 20% of the predicted value is often considered the upper limit of the normal range. An increase in FEV1 loss from a median value of −10% (stratum 1) to even as little as 9% of the predicted value (stratum 2) was associated with increased risk of lung cancer after adjusting for smoking exposure. Among the 153 patients assigned to stratum 1, 18.3% were ever smokers, and 7.2% had lung cancer. Stratum 1 membership does not imply that no smoking-related FEV1 loss had occurred because the pre-exposure values of FEV1 loss were not available. Our data only allowed for a single comparison of an individual’s FEV1 loss with the external predicted values. Even an FEV1 loss < 0% of the predicted value may be significantly higher than this individual’s true normal value.

The predicted values for lung cancer risk lie within the range of [0–1]. A patient with a risk close to 1 due to a high smoking exposure is less able to increase his or her FEV1 loss-related risk when moving from the 5^th^ (−22% of the predicted value) to the 95^th^ percentile (56% of the predicted value) of this variable than a never smoker. This limitation of Δ gives Δ_norm_ an advantage as an outcome variable. In a patient with a smoking exposure of 70 pack years who moved from the 5^th^ to the 95^th^ percentile of FEV1 loss, the lung cancer risk increased by 0.34, which was approximately two-fold higher than the increase observed in never smokers (Δ = 0.16). The values for Δ_norm_ showed an almost linear increase up to approximately 70 pack years and then levelled off without reaching a plateau up to an exposure of 200 pack years. Our data are inadequate to obtain a firm conclusion on this point because only 21 patients reported a smoking exposure above 70 pack years, 11 of whom had lung cancer. Furthermore, the effect of smoking exposure on lung cancer risk becomes so dominant at ≥ 70 pack years that the impact of FEV1 loss as an additional predictive variable is difficult to recognize.

Current guidelines recommend criteria for selecting individuals without symptoms and signs of lung cancer for screening using low-dose helical computed tomography. These criteria include patients *(i)* 50 years of age or older *(ii)* with a ≥ 20 pack-year history of smoking and *(iii)* the presence of at least one additional risk factor, such as radon exposure, occupational exposure, a cancer history, a family history of lung cancer in first-degree relatives, pulmonary fibrosis, or COPD [14]. In this study, an FEV1 loss > 20% of the predicted value was associated with increased odds of lung cancer among smokers with a ≥ 10 pack-year history. Thus, in addition to calculating the number of pack years, measurements of FEV1 loss are useful as a diagnostic tool to identify smokers who are facing a particularly high risk of developing lung cancer.

Our data cannot determine whether FEV1 loss is a direct driving factor in lung carcinogenesis. We presume that FEV1 loss is more of a surrogate marker of the carcinogenic damage and the extent of disease progression rather than a direct participant in the underlying disease process.

The prevalence values for lung cancer and COPD were 40 and 44% among ever smokers, respectively. Epidemiological studies of the general population have shown that the majority of ever smokers do not develop lung cancer or COPD. The lifetime risk of lung cancer is only 15% among ever smokers. Approximately 50–80% of patients with lung cancer have COPD. Although the traditional view has been that the prevalence of COPD among ever smokers is only 20–30% [15], one study suggested that as many as one in two smokers may eventually develop COPD, provided that they live long enough [16]. The inclusion criteria of our study are crucial for interpreting the results. The presence of both a previous or active cancer and cardiac or pulmonary symptoms were prerequisites for inclusion. The cohort did not include any healthy smokers. A smoker who was less susceptible to developing COPD was more likely to be asymptomatic and thus less likely to be included in this study than a COPD-susceptible smoker. Hence, our findings overestimate the strength of the relationship between smoking exposure and FEV1 loss, particularly in patients with other cancers. This referral bias does not invalidate the main finding of our study. Rather, the study design provides insights into the relationship between smoking exposure and FEV1 loss in determining lung cancer risk as if it were viewed under a magnifying glass.

We included patients with asthma without evidence of airway obstruction and did not exclude patients with restrictive ventilatory defects. As shown in Table 1, the FEV1 loss strata 1 and 2 were well balanced with respect to patients with asthma, whereas patients with restrictive ventilatory defects were not evenly distributed across the three strata of FEV1 loss. There is no epidemiologic evidence of an association between asthma and lung cancer [17], and none of our information suggests that the inclusion of patients with asthma introduced any bias. In patients with restrictive ventilatory defects, the FEV1 % predicted values were adjusted for the VC % predicted values instead of the TLC % predicted values if the VC % predicted value was greater than TLC % predicted value [18]. This adjustment was made *(i)* because the VC % predicted value is less subject to measurement error than the TLC % predicted value and *(ii)* to maintain a minimum difference between the measured and adjusted FEV1 % predicted values.

The time interval between cancer diagnosis and lung function testing was > 1 year in 27% of patients with lung cancer and in 84% of patients with other cancers. Thus, the values for FEV1 loss differed significantly at the two time points in a number of these patients. The rate of decrease in FEV1 varies, with increasing rates observed among current smokers and patients with bronchodilator reversibility or emphysema [19]. Therefore, we refrained from estimating the FEV1 loss at the time of cancer diagnosis by extrapolating backwards in time from the FEV1 loss observed at the time of referral and used the latter value for the statistical analyses.

Direct markers of airway inflammation were not available. The serum CRP level has gained acceptance as an indirect and quantitative marker of airway inflammation. In patients with stable COPD, the serum CRP levels are positively correlated both with FEV1 loss and an accelerated rate of decrease in FEV1 over time [20–23]. If ln (CRP) were only a surrogate marker of FEV1 loss, the second adjustment of ln (CRP) would be expected to equalize the difference in ln (CRP) between patients with lung cancer and patients with other cancers, but this hypothesis was not confirmed. The values for ln (CRP) remained significantly higher in patients with lung cancer after adjusting for FEV1 loss. These findings are consistent with the hypothesis that airway inflammation exerts a unique role in determining the patient’s risk of developing lung cancer. Because our data are cross-sectional, no causal relationships can be established. Rather, the results of this study are purely descriptive and provide no insights into the time course and interactions by which smoking exposure, FEV1 loss and inflammation influence the development of lung cancer.

This single-center study has further limitations. First, the data on smoking exposure were collected retrospectively. Many patients with symptomatic COPD or lung cancer feel guilty about smoking. Patients with lung cancer or symptomatic COPD who report no or a low smoking exposure might thus have systematically underreported their smoking exposure compared to patients without smoking-related lung disease. If this kind of reporting bias had occurred, the true associations of smoking exposure with lung cancer risk and FEV1 loss were stronger than the values we estimated. We cannot discount this possibility; however, we found no support in the literature for this assumption. Second, the smoking status at the time of referral was not objectively validated. Third, other risk factors for lung cancer – e.g., a family history of lung cancer, the presence of pulmonary fibrosis and exposure to second-hand smoke, radon, gas, asbestos and other substances known to cause lung cancer – were not included in our risk prognosis model due to a paucity of and a lack of data. Fourth, the number of patients with lung cancer was too low to determine sex- and age-specific risks and to differentiate between histological types of lung cancer [24–26]. Fifth, the scope of this study was limited to FEV1 loss and did not address the role of emphysema in determining the patient’s predicted risk of lung cancer. Sixth, we did not exclude patients with coexisting acute infections or chronic non-malignant inflammatory disorders nor did we adjust the ln (CRP) values for these conditions. Hence, the background noise that influenced the CRP level was high. This limitation tends to dilute the effect of inflammation in the lung on the CRP level and therefore does not weaken the strength of our findings. Finally, the 368 patients with other cancers constitute a heterogeneous cohort that is specific to this institution. Our findings are not generalizable to other populations.

## Conclusion

FEV1 loss is a predictor of lung cancer risk. The effect of FEV1 loss on this outcome increases with the level of previous smoking exposure. In addition to calculating the number of pack years, measurements of FEV1 loss are a useful method to identify patients facing an increased risk of developing lung cancer.

## Abbreviations

BMI: Body mass index
CI: Confidence interval
COPD: Chronic obstructive pulmonary disease
CRP: C-reactive protein
eGFR: Estimated glomerular filtration rate
FEV1: Forced expiratory volume in 1 s
OR: Odds ratio
TLC: Total lung capacity
VC: Vital capacity
